# Policosanol from Insect Wax Attenuates Atherosclerosis in Mice

**DOI:** 10.3390/foods15122109

**Published:** 2026-06-11

**Authors:** Xian Li, Chenjing Ma, Xin Zhang, Hang Chen, Ying Feng, Xiaoming Chen

**Affiliations:** 1 Institute of Highland Forest Science, Chinese Academy of Forestry, Kunming 650224, China; lixian@caf.ac.cn (X.L.); machenjing@caf.ac.cn (C.M.); stuchen6481@gmail.com (H.C.); yingf@hotmail.com (Y.F.); cafcxm@139.com (X.C.); 2 Yunnan Key Laboratory of Breeding and Utilization of Resource Insects, Kunming 650224, China; 3 Key Laboratory of Protection and Utilization of Insects, National Forestry and Grassland Administration, Kunming 650224, China

**Keywords:** policosanol from insect wax, atherosclerosis, ApoE^−/−^ mice, dyslipidemia, low-grade inflammation, LOX-1/NF-κB

## Abstract

Aging-associated dyslipidemia and chronic low-grade inflammation contribute to atherosclerosis and cardiovascular risk. As a blend of long-chain aliphatic alcohols, policosanol from insect wax (PIW) has been documented to regulate lipid metabolism. However, the effects of PIW on atherosclerosis remain insufficiently characterized. In this study, ApoE^−/−^ mice fed a high-fat diet were concurrently administered PIW (75 and 150 mg/kg) for eight weeks. PIW was associated with weight gain reduction and improvement in lipid profile, particularly a decrease in triglycerides and total cholesterol. PIW also lowered circulating inflammatory biomarkers (IL-6, TNF-α, and C-reactive protein). Histopathological analyses revealed attenuated hepatic injury and reduced aortic lipid deposition and lesion features. In parallel, PIW reduced serum endothelin-1 and oxidized LDL levels and modulated aortic ET-1, MMP-9/TIMP-1 balance, and LOX-1/NF-κB-related protein signals. Notably, as PIW was administered concurrently with high-fat diet induction, these findings should be interpreted within a preventive intervention framework. Collectively, PIW help attenuate HFD-associated atherosclerotic features and hold promise as a functional food ingredient for cardiovascular health and healthy aging.

## 1. Introduction

Aging is implicated in the development of several chronic diseases, including cancer, diabetes, cardiovascular diseases, and neurodegenerative disorders [1], and age-related vascular alterations are closely involved in atherosclerosis, a key pathological contributor to cardiovascular morbidity and mortality [2,3]. Recent research indicates that vascular tissue ages comparatively early and is especially vulnerable to age-associated alterations [4]. Therefore, vascular health is crucial for combating aging. Importantly, atherosclerosis is considered not only a vascular disease but also a systemic cardiometabolic disorder associated with aging, which causes immense harm to the health of the elderly and imposes a substantial burden on society.

The various physiological changes caused by aging often simultaneously lead to the development of neurodegenerative diseases, atherosclerosis, and vascular dementia, suggesting that these diseases may share some common characteristics in their pathogenic mechanisms and treatment strategies. Studies have shown that age-related lipid metabolism disorders and low-grade chronic inflammation are common mechanisms linking atherosclerosis and neurodegenerative diseases [5,6,7,8,9]. Chronic stress is also considered an important contributing factor in the progression of neurodegenerative diseases and atherosclerosis. Moreover, dysfunction of Chaperone-mediated autophagy (CMA) may be involved in the development of neurodegenerative disorders, tumors, and atherosclerosis [10]. Chronological aging causes subtle changes in the neuronal structure and function of specific neuronal circuits, which trigger chronic inflammation [1]. Meanwhile, inflammation is an important driver of atherosclerosis. Inflammation, neuroinflammation, and the impairment of resolution of inflammation are crucial pathogenic hallmarks in different neurodegenerative disorders, including Alzheimer’s disease and Parkinson’s disease [11,12,13]. In our preliminary studies, we found that Policosanol from Insect Wax (PIW) had a mitigating effect on the symptoms of neurodegenerative models of *Caenorhabditis elegans* [14], suggesting that PIW may have the potential to alleviate atherosclerosis.

Policosanol refers to a naturally occurring blend of saturated long-chain aliphatic alcohols, with tetracosanol, hexacosanol, octacosanol, and triacontanol as its principal constituents, and can be obtained from natural sources such as beeswax, sugarcane wax, rice bran wax, white wax, and corn bran wax [15]. Hexacosanol and octacosanol have been widely investigated for their potential roles in lipid regulation, oxidative stress reduction, inflammation suppression, and endothelial protection [16,17,18], and products containing these compounds are already available on the market. Among the various sources of higher policosanol, insect waxes contain a higher proportion of hexacosanol and octacosanol compared to other sources, approximately three times higher than in sugarcane wax and rice bran wax [19]. Meanwhile, policosanol can also be used as a mixture which is more cost-effective. However, the efficacy of policosanols depends on the purity and composition of the preparation [20].

Through a reduction method, insect wax can be effectively converted into high-purity PIW, which mainly comprises tetracosanol (C_24_H_49_OH), hexacosanol (C_26_H_53_OH), octacosanol (C_28_H_57_OH), and triacontanol (C_30_H_61_OH) [14]. Insect wax secreted by *Ericerus pela* Chavannes (Hemiptera: Coccidae) has been used in China for over a thousand years as both a traditional medicine and an edible material [14]. Notably, *Ericerus pela* wax is still consumed as an edible material in certain local practices in Hunan Province, China, where it is traditionally believed to confer health benefits. Toxicological evaluations have suggested that PIW has a favorable safety profile for oral intake, supporting its potential application in functional foods [21].

Our previous work indicated that PIW exerts neuroprotective effects in Caenorhabditis elegans models of Alzheimer’s and Parkinson’s diseases and improves cognitive impairment in mouse models. PIW also ameliorated lipid metabolic disturbances in high-fat diet-fed rats, supporting its broader role in metabolic regulation [18]. These findings suggest that PIW may target shared mechanisms underlying aging-related chronic diseases. Given the close mechanistic links between dyslipidemia, chronic inflammation, neurodegeneration, and atherosclerosis, the present study aimed to investigate the effects of PIW on atherosclerosis progression using ApoE^−/−^ mice fed a high-fat diet. By evaluating serum lipid levels, inflammatory markers, and aortic pathological changes, we sought to assess the potential of PIW as a functional food ingredient for the dietary management of atherosclerosis and healthy aging.

## 2. Materials and Methods

### 2.1. Policosanol

Policosanol was prepared from insect wax obtained from Zhijiang County (Huaihua, China) using a lithium aluminum hydride-mediated reduction protocol, as previously described [14,22]. The component concentrations are expressed as mass concentration (mg/L) in the purified extract, as determined by gas chromatography–mass spectrometry (GC-MS) following saponification and silylation, consistent with previously described methods [14]. The composition of the PIW was characterized by the presence of four major long-chain aliphatic alcohols: tetracosanol (C_24_H_49_OH), hexacosanol (C_26_H_53_OH), octacosanol (C_28_H_57_OH), and triacontanol (C_30_H_61_OH) at concentrations of 170.6 mg/L, 644.4 mg/L, 515.5 mg/L, and 170.9 mg/L, respectively [14]. Based on preliminary experiments, rats fed a high-fat diet and treated orally with 75 mg/kg and 150 mg/kg PIW exhibited lower serum total cholesterol levels compared with those receiving other tested doses of PIW. Accordingly, these two doses were selected for subsequent experiments. PIW suspensions were prepared in 0.5% sodium carboxymethyl cellulose (CMC-Na; C8620-25g; Beijing Solarbio Science & Technology Co., Ltd., Beijing, China) at concentrations corresponding to the low-dose (75 mg/kg) and high-dose (150 mg/kg) formulations.

### 2.2. Experimental Animals

A total of 60 healthy male apolipoprotein E-deficient (ApoE^−/−^) mice on a C57BL/6J background, 8 weeks old and weighing 20–25 g, were obtained from Changzhou Cavens Laboratory Animal Co., Ltd.(Changzhou, Jiangsu, China) (specific-pathogen-free grade; license number SCXK(Su)2021–0013). The animals were housed under standard laboratory conditions with a temperature of 23–25 °C, relative humidity of 50 ± 10%, and a 12 h light/12 h dark cycle, with ad libitum access to food and water. Prior to the experiments, the mice were acclimated for one week. The study adhered to the ARRIVE guidelines and to Directive 2010/63/EU throughout, with ethical approval obtained from Ethics Committee of Institute of Highland Forest Science.

### 2.3. Experimental Treatment

The mice were stratified by body weight, and then randomly assigned to five experimental groups using a random number table, including the normal Control group, the Model group, the positive control group, the low-dose PIW group, and the high-dose PIW group. Each group contained 12 mice. Group allocation and all experimental procedures were conducted by blinded personnel to reduce bias. To minimize potential confounders, cage positions were regularly rotated to avoid location-related effects such as differences in light exposure or environmental conditions. In addition, treatments and measurements were performed in a randomized and balanced order to minimize potential order effects. Mice in the normal Control group were fed a standard chow diet (Jiangsu Synergy Bioengineering Co., Ltd., Nanjing, Jiangsu, China), while those in the remaining groups were fed a high-fat diet (Beijing Botai Hongda Biotechnology Co., Ltd., Beijing, China; catalog no. HD0012a) for 8 weeks to induce atherosclerosis. All animals had ad libitum access to food and water throughout the experimental period.

During the model induction period, mice in the PIW low- and high-dose groups received daily oral gavage of PIW suspensions prepared in 0.5% CMC-Na at concentrations of 75 mg/kg and 150 mg/kg, respectively. The positive Control group was administered atorvastatin calcium (Lipitor^®^, Pfizer Inc., New York, NY, USA) at a dose of 10 mg/kg body weight, formulated in the same vehicle. Mice in the normal Control and Model groups received an equivalent volume of vehicle alone. All treatments were given by daily oral gavage for 8 weeks, with the administration volume adjusted to 0.2 mL per 10 g of body weight.

### 2.4. Measurement of Body Weight and Feed Intake

During the experimental period, the general growth status of the mice was monitored continuously. Body weight was measured and recorded individually for each mouse on a weekly basis. The gavage volume was adjusted once per week according to changes in body weight to ensure accurate dose administration. Food consumption was assessed every two weeks by recording the feed intake of each group.

### 2.5. Measurement of Serum Lipid Parameters

One week after the start of the experiment, six mice were randomly selected from each group following an overnight fast, and blood samples were collected from the retro-orbital venous plexus. Place the blood on ice for 30 min to allow clotting, then centrifuge at 4000× *g* for 10 min at 4 °C. Aliquot the resulting serum and store at −80 °C. The same animals were subsequently sampled at weeks 2, 4, and 8 using the same procedure to obtain longitudinal serum samples. After completion of all collections, serum levels of total cholesterol (T-CHO), triglycerides (TGs), low-density lipoprotein cholesterol (LDL-C), and high-density lipoprotein cholesterol (HDL-C) were measured simultaneously using commercial assay kits (Nanjing Jiancheng Bioengineering Institute, China; T-CHO: A111-1-1, TG: A110-1-1, LDL-C: A113-1-1, HDL-C: A112-1-1) on a Varioskan LUX multifunctional microplate reader (Thermo Fisher Scientific, Waltham, MA, USA) according to the manufacturers’ instructions.

### 2.6. Determination of Serum Inflammatory Cytokines

Following an overnight fast at week 1, six mice from each group were selected and blood samples were collected via the retro-orbital venous plexus. Samples were kept on ice for 30 min to permit clotting, after which serum was separated by centrifugation (4000× *g*, 4 °C, 10 min), divided into aliquots, and kept at −80 °C until analysis. The same mice were sampled again at weeks 4, 6, and 8 using the same procedure to provide longitudinal serum samples. After completion of all collections, commercial ELISA kits were used to measure serum inflammatory markers according to the manufacturer’s instructions, including interleukin-6 (IL-6; Mouse IL-6 ELISA Kit, MultiSciences Biotech Co., Ltd., Hangzhou, China; Cat# EK206), keratinocyte-derived chemokine (KC/CXCL1; IL-8–axis–related chemokine readout measured using a QuantiCyto^®^ Mouse KC (IL-8) ELISA kit, XinboSheng Biotech Co., Ltd. (Neobioscience), Beijing, China; Cat# EMC104), tumor necrosis factor-α (TNF-α; Mouse TNF-α ELISA Kit, MultiSciences Biotech Co., Ltd., Hangzhou, China; Cat# EK282), and C-reactive protein (CRP; QuantiCyto^®^ Mouse CRP ELISA kit, XinboSheng Biotech Co., Ltd. (Neobioscience), Beijing, China; Cat# EMC028).

### 2.7. Histopathological Analysis of Liver and Aorta Tissues

Following the 8-week preventive treatment, a random sample of three mice from every group was subjected to a 4 h fast, and euthanized by carbon dioxide. The liver and thoracic aorta were then rapidly and carefully excised for histological analysis. For liver histology, tissues were fixed in 4% paraformaldehyde for 24 h, dehydrated, embedded in paraffin, and sectioned at 4 μm thickness. The sections were stained with hematoxylin and eosin (H&E) using H&E staining solution (G1003, Wuhan Servicebio Technology Co., Ltd., Wuhan, China), and hepatocyte morphology and pathological changes were evaluated under a light microscope (Nikon, E100, Tokyo, Japan) equipped with a digital imaging system (Nikon, DS-U3, Tokyo, Japan).

For aortic histology, the proximal segment of the aorta was collected from the aortic arch, quickly rinsed at 4 °C physiological saline to remove blood, and two to three 5 mm aortic rings were excised. After fixation in 4% paraformaldehyde, the rings were paraffin-embedded and cut into 4-μm sections. H&E staining was performed to examine the structure of the aortic wall, including the intima, media, and adventitia, as well as indicators of atherosclerotic lesion formation, such as intimal thickening and smooth muscle cell proliferation. To enable semiquantitative and objective histopathological assessment, all sections were evaluated by two independent blinded observers. Aortic atherosclerotic lesions were graded on a 0–4 scale: 0 = no lesion; 1 = focal intimal thickening without lipid deposition; 2 = fatty streaks with moderate foam cell infiltration; 3 = established fibroatheroma with fibrous cap; 4 = complex lesion with necrotic core or calcification. Hepatic injury was evaluated using the NAFLD Activity Score (NAS), incorporating steatosis (0–3), lobular inflammation (0–3), and hepatocellular ballooning (0–2). For each animal, five randomly selected microscopic fields were scored, and the mean value was used for statistical analysis (n = 3 animals per group). Scoring criteria were adapted from published methods [23,24].

To assess lipid deposition in the aorta, following the collection of aortic rings, the remaining aorta was carefully cleared of blood, wrapped in pre-cooled aluminum foil, rapidly frozen in liquid nitrogen, and stored at −80 °C. Lipid-rich atherosclerotic plaques were visualized by staining with Oil Red O (G1016, Wuhan Servicebio Technology Co., Ltd., Wuhan, China).

### 2.8. Inflammatory Marker and Protein Expression Analyses in Serum and Aorta

At the end of the experiment, the remaining aorta was excised up to the bifurcation of the abdominal aorta, rinsed with ice-cold physiological saline to remove excess fat and blood, and blotted dry with filter paper. Three mice were included per experimental group for this procedure. Portions of the tissue were weighed and homogenized in ice-cold physiological saline at a ratio of 1:9 (*w*/*v*). Following centrifugation at 3000 rpm and 4 °C for 20 min, the collected supernatants were assayed for endothelin-1 (ET-1), tissue inhibitor of metalloproteinases-1 (TIMP-1), and matrix metalloproteinase-9 (MMP-9) with commercial ELISA kits (ET-1: H093; Nanjing Jiancheng Bioengineering Institute, Nanjing, China; TIMP-1: Mouse TIMP-1 ELISA Kit, Beyotime Biotechnology, Shanghai, China; Cat# PT883; MMP-9: Mouse MMP-9 ELISA Kit, Beyotime Biotechnology, Shanghai, China; Cat# PM733). In addition, serum collected at week 8 was analyzed for ET-1 and oxidized low-density lipoprotein (ox-LDL) using corresponding ELISA kits (ET-1: H093; ox-LDL: H248; Nanjing Jiancheng Bioengineering Institute, Nanjing, China).

Another portion of the aortic tissue was stored at −80 °C for Western blot (WB) analysis. At the time of analysis, tissues were weighed and homogenized on ice in RIPA lysis buffer containing PMSF. After centrifugation at 12,000 rpm for 5 min at 4 °C, the resulting supernatants were used for BCA-based protein concentration determination. Sample buffer was added to prepare the protein samples, and 20 μg of protein per lane was loaded. Equal amounts of protein from three biologically independent samples per group were loaded. Proteins were separated by 10% SDS-PAGE and subsequently transferred onto PVDF membranes. After a brief wash with TBST, the membranes were blocked in TBST containing 5% skim milk for 1 h at room temperature. Primary antibodies were diluted and used according to the manufacturers’ instructions (anti-lectin-like oxidized low-density lipoprotein receptor-1 [LOX-1], Abcam, Cat# ab214427, 1:1000; anti–NF-κB p65, Abcam, Cat# ab16502, 1:1000; and anti–β-actin, HuaAn Biotechnology, Hangzhou, China, Cat# ET1702-67, 1:3000), and incubated overnight at 4 °C. Membranes were washed three times with TBST (5 min each) and then incubated with an HRP-conjugated goat anti-rabbit IgG (H + L) secondary antibody (Beyotime Biotechnology, Shanghai, China; Cat# A0208; 1:1000) for 1 h at room temperature. After four additional washes with TBST (5 min each), protein bands were detected using BeyoECL Plus enhanced chemiluminescence reagents (Beyotime Biotechnology, Shanghai, China; Cat# P0018S) and visualized through chemiluminescence imaging. β-Actin served as the loading control, and band intensities were analyzed with ImageJ software version 1.52a (National Institutes of Health, Bethesda, MD, USA).

### 2.9. Statistical Analysis

All quantitative data are presented as mean ± standard error of the mean (SEM). Statistical analyses and graph generation were performed in GraphPad Prism (version 10.5.0). For comparisons between groups, either one-way ANOVA or a nonparametric test was applied, depending on whether the data satisfied normality assumptions. Effect sizes were calculated using Cohen’s d (for two-group comparisons) or η^2^ (for ANOVA), and 95% confidence intervals for the effect sizes were reported. *p* values < 0.05 were considered statistically significant.

## 3. Results

### 3.1. PIW Reduced Body-Weight Gain in ApoE^−/−^ Mice

**Figure 1:**
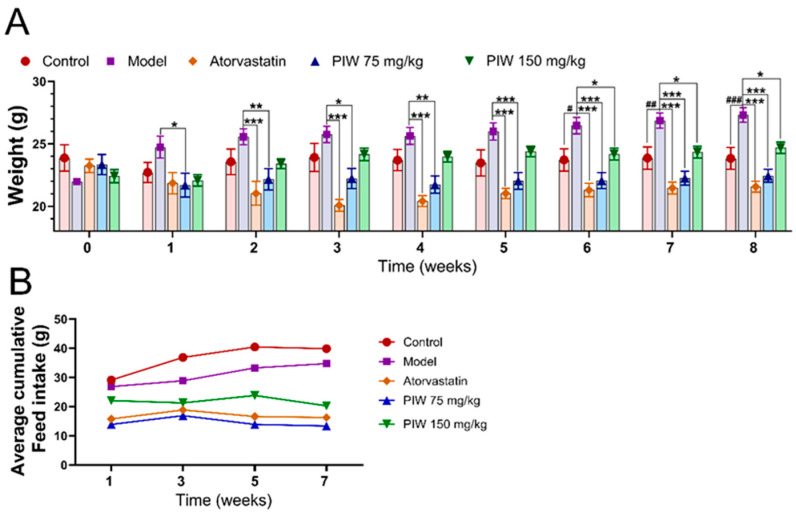
Effects of PIW on body weight and cumulative feed intake in ApoE^−/−^ mice: (**A**) Weekly body weight from week 0 to week 8 in ApoE^−/−^ mice in the Control, Model, atorvastatin, PIW 75 mg/kg, and PIW 150 mg/kg groups. Data are presented as mean ± SEM (n = 12). ^#^ *p* < 0.05, ^##^ *p* < 0.01, ^###^ *p* < 0.001, compared with the Control group; * *p* < 0.05, ** *p* < 0.01, *** *p* < 0.001, compared with the Model group. (**B**) Average cumulative feed intake over the experimental period for each treatment group.

We continuously monitored body weight (weeks 0–8) and average cumulative feed intake during PIW administration in ApoE^−/−^ mice (Figure 1A,B). Body weight in the model group rose progressively relative to the Control group, reaching statistical significance during weeks 6–8 (*p* < 0.05, *p* < 0.01, *p* < 0.001, respectively) (Figure 1A). Relative to the model group, body weight was significantly reduced after treatment with atorvastatin or PIW: atorvastatin produced a marked decrease from weeks 2–8 (*p* < 0.001), whereas 75 mg/kg PIW significantly reduced body weight from weeks 1–8 (*p* < 0.05, *p* < 0.01, *p* < 0.001, respectively). In contrast, 150 mg/kg PIW led to a significant reduction in body weight only during weeks 6–8 (*p* < 0.05) (Figure 1A). For average cumulative feed intake, values in both the control and Model groups increased progressively over time. In contrast, cumulative intake in the atorvastatin group and the PIW-treated groups (75 mg/kg and 150 mg/kg) showed a slower increase over time. Notably, cumulative intake in the atorvastatin group and the 75 mg/kg PIW group remained relatively stable from weeks 3–7 (Figure 1B).

### 3.2. PIW Ameliorated Blood Lipid Profiles in ApoE^−/−^ Mice

**Figure 2:**
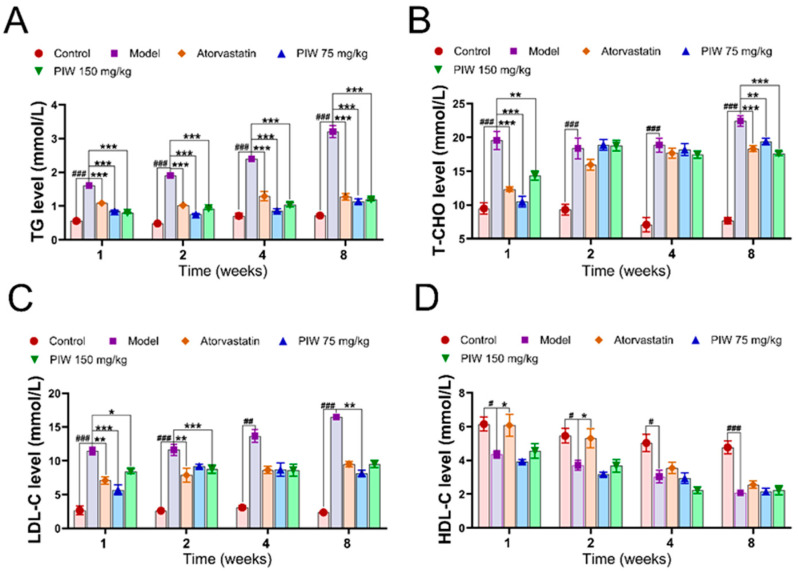
Effects of PIW on serum lipid profiles in ApoE^−/−^ mice: (**A**) Serum TG levels at weeks 1, 2, 4, and 8. (**B**) Serum T-CHO levels at weeks 1, 2, 4, and 8. (**C**) Serum LDL-C levels at weeks 1, 2, 4, and 8. (**D**) Serum HDL-C levels at weeks 1, 2, 4, and 8. Data are presented as mean ± SEM (n = 6). ^#^ *p* < 0.05, ^##^ *p* < 0.01, ^###^ *p* < 0.001, compared with the Control group; * *p* < 0.05, ** *p* < 0.01, *** *p* < 0.001, compared with the Model group.

Over 1–8 weeks of continuous PIW treatment, we tracked serum levels of triglyceride (TG), total cholesterol (T-CHO), low-density lipoprotein cholesterol (LDL-C), and high-density lipoprotein cholesterol (HDL-C) in ApoE^−/−^ mice (Figure 2A–D). Compared with the Control group, the Model group displayed a sustained dyslipidemic phenotype over the experimental period, characterized by significantly elevated TG, T-CHO, and LDL-C levels (*p* < 0.01 and *p* < 0.001, respectively), whereas the HDL-C level was significantly decreased (*p* < 0.05 and *p* < 0.001, respectively) (Figure 2A–D). Compared with the model group, both atorvastatin and PIW treatment (75 mg/kg and 150 mg/kg) significantly reduced TG levels at weeks 1–8 (*p* < 0.001) (Figure 2A). In addition, atorvastatin and PIW (75 mg/kg and 150 mg/kg) produced significant reductions in T-CHO at weeks 1 and 8 (Figure 2B). For LDL-C, significant decreases were observed at week 1 in the atorvastatin and PIW groups (*p* < 0.05, *p* < 0.01, and *p* < 0.001, respectively); a significant decline at week 8 was confined to the 75 mg/kg PIW group (*p* < 0.001) (Figure 2C). For HDL-C, a significant increase was detected only in the atorvastatin group during weeks 1–2 (*p* < 0.001) (Figure 2D).

### 3.3. PIW Altered Serum Inflammatory Biomarker Levels in ApoE^−/−^ Mice

**Figure 3:**
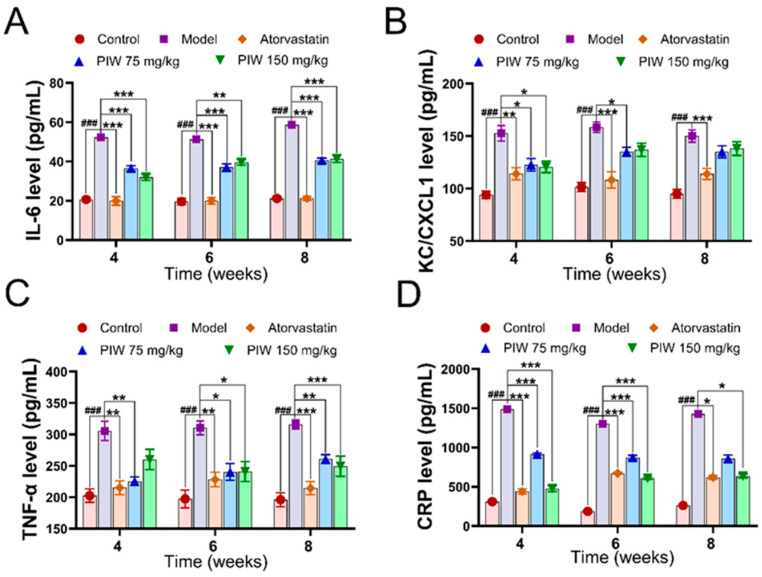
Effects of PIW on serum inflammatory markers in ApoE^−/−^ mice: (**A**) Serum IL-6 levels measured at weeks 4, 6, and 8 in ApoE^−/−^ mice in the Control, Model, atorvastatin, PIW 75 mg/kg, and PIW 150 mg/kg groups. (**B**) Serum keratinocyte-derived chemokine levels (KC/CXCL1; IL-8–axis-related chemokine readout) measured at weeks 4, 6, and 8 in the indicated groups. (**C**) Serum TNF-α levels measured at weeks 4, 6, and 8 in the indicated groups. (**D**) Serum CRP levels measured in weeks 4, 6, and 8 in the indicated groups. Data are presented as mean ± SEM (n = 6). ^###^ *p* < 0.001, compared with the Control group; * *p* < 0.05, ** *p* < 0.01, and *** *p* < 0.001, compared with the Model group.

We further assessed serum IL-6, KC/CXCL1 (IL-8–axis–related chemokine readout), TNF-α, and C-reactive protein (CRP) levels in ApoE^−/−^ mice during weeks 4 to 8 following PIW administration. Throughout this period, the Model group showed significantly higher levels of IL-6, KC/CXCL1, TNF-α, and CRP than the Control group (*p* < 0.001) (Figure 3A–D). Compared with the model group, atorvastatin and PIW treatment (75 mg/kg and 150 mg/kg) significantly reduced KC/CXCL1 and CRP levels at week 4 (*p* < 0.05, *p* < 0.01, and *p* < 0.001, respectively), and significantly decreased serum IL-6 and TNF-α levels at weeks 6–8 (*p* < 0.05, *p* < 0.01, and *p* < 0.001, respectively) (Figure 3A–D). In addition, atorvastatin significantly lowered KC/CXCL1 and CRP levels at weeks 6–8 (*p* < 0.05 and *p* < 0.001, respectively) (Figure 3B,D). The 75 mg/kg PIW group showed significant reductions in KC/CXCL1 and CRP at week 6 (*p* < 0.05 and *p* < 0.001, respectively) (Figure 3B,D), whereas 150 mg/kg PIW significantly decreased CRP levels at weeks 6–8 (*p* < 0.05 and *p* < 0.001, respectively) (Figure 3D).

### 3.4. PIW Attenuated Liver and Aortic Histopathological Changes in ApoE^−/−^ Mice

**Figure 4:**
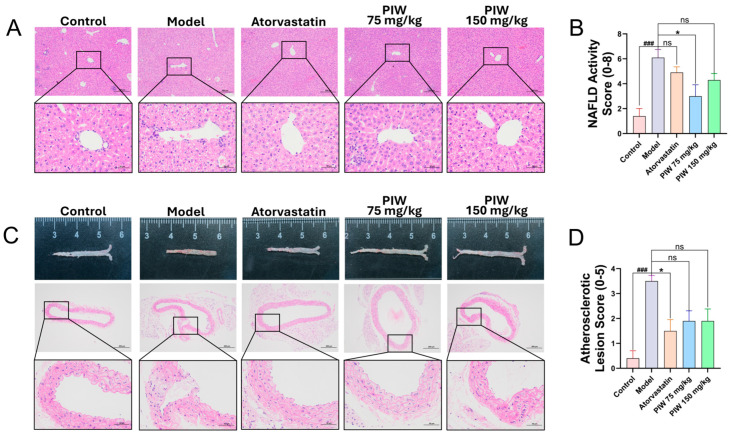
PIW attenuated liver and aortic histopathological changes in ApoE^−/−^ mice: (**A**) Representative histological images of liver tissue (400×). (**B**) Quantification of the NAFLD activity score based on H&E-stained liver sections. (**C**) Representative histological images of aortic tissue (400×). (**D**) Quantification of the atherosclerotic lesion score based on H&E-stained aortic sections. Data are presented as mean ± SEM (n = 5). ^###^ *p* < 0.001, compared with the Control group; * *p* < 0.05, compared with the Model group; ns, not significant.

H&E staining revealed that livers from Control ApoE^−/−^ mice maintained well-preserved hepatic architecture, with hepatocytes exhibiting clear cellular morphology and distinct nuclear features (Figure 4A). In contrast, the Model group showed prominent histopathological changes, characterized by hepatocellular swelling, less distinct cell boundaries, and vacuole-like changes in the perinuclear region (Figure 4A). In the atorvastatin group, mild hepatocellular swelling was also observed; however, perinuclear vacuole-like changes appeared less pronounced than in the Model group (Figure 4A). In the 75 mg/kg PIW group, hepatic histological features were relatively preserved, with clearer cellular outlines, although widened intercellular spaces were noted (Figure 4A). In the 150 mg/kg PIW group, hepatocellular swelling and perinuclear vacuole-like changes remained evident, but cellular contours appeared more clearly delineated than in the Model group (Figure 4A). Histopathological scoring of liver sections showed that the NAFLD activity score was significantly increased in the Model group compared with the Control group (*p* < 0.001) (Figure 4B). Compared with the Model group, the 75 mg/kg PIW group showed a significant reduction in the NAFLD activity score (*p* < 0.05) (Figure 4B). The atorvastatin group and the 150 mg/kg PIW group also showed a decreasing trend in the NAFLD activity score; however, the differences were not statistically significant (Figure 4B).

Oil Red O and H&E staining of aortic tissues showed that the Control group exhibited a smooth vessel wall surface with well-preserved overall morphology. H&E staining showed a well-organized lamellar structure of the vessel wall without overt disruption (Figure 4C). In the Model group, Oil Red O staining revealed numerous red-positive areas consisting of increased lipid deposition, accompanied by irregularities and protrusion-like changes on the vessel wall surface. H&E staining further indicated disruption of aortic wall architecture with reduced integrity of the lamellar organization (Figure 4C). In the atorvastatin and PIW-treated groups (75 mg/kg and 150 mg/kg), Oil Red O–positive areas and structural alterations were still evident. However, lesion-related features appeared less extensive than in the Model group. Consistently, H&E staining showed a relatively better-preserved vessel wall with identifiable lamellar organization, although focal areas of distortion and disorganization remained (Figure 4C). Consistent with these histological observations, scoring of aortic pathological injury showed that the atherosclerotic lesion score was significantly increased in the Model group compared with the Control group (*p* < 0.001) (Figure 4D). The atorvastatin group showed a significant decrease in atherosclerotic lesion score versus the Model group (*p* < 0.05) (Figure 4D). The 75 mg/kg PIW group and the 150 mg/kg PIW group also showed a decreasing trend in the atherosclerotic lesion score; however, the differences were not statistically significant (Figure 4D).

### 3.5. PIW Attenuated Vascular Injury-Related Biomarkers in ApoE^−/−^ Mice

**Figure 5:**
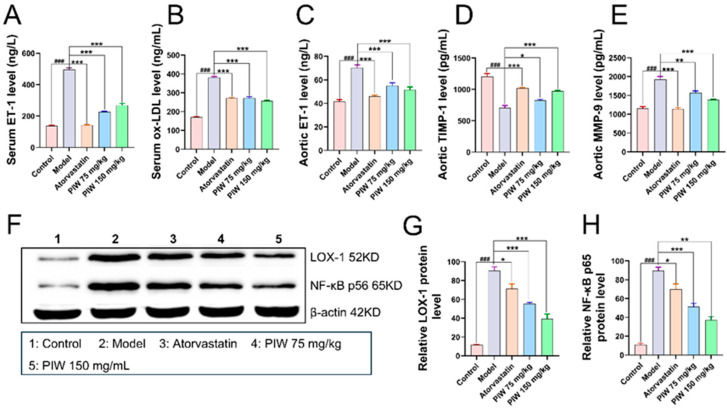
Effects of PIW on serum and aortic inflammatory biomarkers and related protein expression in ApoE^−/−^ mice: (**A**) serum endothelin-1 (ET-1) levels (ng/L); (**B**) serum oxidized low-density lipoprotein (ox-LDL) levels (ng/mL); (**C**) aortic ET-1 levels (ng/L); (**D**) aortic tissue inhibitor of metalloproteinases-1 (TIMP-1) levels (pg/mL); (**E**) aortic matrix metalloproteinase-9 (MMP-9) levels (pg/mL). (**F**) Representative Western blot bands of LOX-1 and NF-κB p65 in aortic tissues, with β-actin as the loading control (lanes: 1, Control; 2, Model; 3, Atorvastatin; 4, PIW 75 mg/kg; 5, PIW 150 mg/kg). (**G**) Relative protein expression level of LOX-1. (**H**) Relative protein expression level of NF-κB p65. Data are presented as mean ± SEM (n = 3). ^###^ *p* < 0.001, compared with the Control group; * *p* < 0.05, ** *p* < 0.01, and *** *p* < 0.001, compared with the Model group.

After 8 weeks of PIW administration, serum endothelin-1 (ET-1) and oxidized low-density lipoprotein (ox-LDL) levels were assessed (Figure 5A,B). Compared with the Control group, the Model group showed significantly elevated serum ET-1 and ox-LDL levels (*p* < 0.001) (Figure 5A,B). Compared with the Model group, atorvastatin and PIW (75 mg/kg and 150 mg/kg) significantly reduced serum ET-1 and ox-LDL levels (*p* < 0.001) (Figure 5A,B). Aortic tissues were then collected to quantify ET-1, tissue inhibitor of metalloproteinases-1 (TIMP-1), and matrix metalloproteinase-9 (MMP-9) levels. Compared with the Control group, the Model group exhibited significantly increased aortic ET-1 and MMP-9 levels (*p* < 0.001), accompanied by a significant decrease in TIMP-1 (*p* < 0.001). Compared with the Model group, atorvastatin and PIW (75 mg/kg and 150 mg/kg) significantly decreased aortic ET-1 and MMP-9 levels (*p* < 0.01 and *p* < 0.001, respectively), and significantly increased TIMP-1 levels (*p* < 0.05 and *p* < 0.001, respectively) (Figure 5C–E). Western blot analysis further showed that LOX-1 and NF-κB p65 protein expression in aortic tissues was markedly upregulated in the Model group compared with the Control group (*p* < 0.001). Conversely, compared with the Model group, atorvastatin and PIW (75 mg/kg and 150 mg/kg) significantly downregulated LOX-1 and NF-κB p65 protein expression (*p* < 0.05, *p* < 0.01, and *p* < 0.001, respectively) (Figure 5F–H).

## 4. Discussion

Atherosclerosis is now widely viewed as a chronic disease with both inflammatory and metabolic components. Its development involves the accumulation of lipids within the arterial wall, dysfunction of the endothelium, and activation of the immune system. Acting in concert, these processes drive the formation and progression of atherosclerotic plaques. In the present study, our results suggest that policosanol from insect wax (PIW) attenuates high-fat diet-induced atherosclerotic phenotypes in ApoE^−/−^ mice, as evidenced by improved serum lipid profiles, reduced systemic and vascular inflammatory markers, alleviated hepatic and aortic histopathological changes, and changes in LOX-1/NF-κB–related protein signals and extracellular matrix remodeling markers. These findings support the potential of PIW as a functional intervention for cardiovascular health and healthy aging.

Dyslipidemia is a core driver of atherogenesis, promoting the infiltration and retention of lipoproteins in the arterial wall and triggering oxidative modifications such as ox-LDL formation. Scavenger receptors, particularly LOX-1, mediate ox-LDL uptake into endothelial cells, smooth muscle cells, and macrophages, thereby initiating endothelial dysfunction and vascular inflammation [25]. LOX-1 activation has been linked to downstream inflammatory signaling events, including NF-κB activation, which can amplify pro-inflammatory gene expression and promote foam cell-related processes [26]. In this study, PIW significantly reduced serum ox-LDL and decreased aortic LOX-1 and NF-κB p65 protein expression, suggesting that PIW may attenuate LOX-1-related inflammatory signaling during diet-induced atherogenesis.

Chronic inflammation is a hallmark of aging and is intricately involved in multiple stages of atherosclerosis. Pro-inflammatory mediators such as IL-6, KC/CXCL1 (a functional analogue of IL-8 in mice), TNF-α, and CRP contribute to endothelial activation, leukocyte recruitment, and plaque progression [27,28,29,30]. The significant reductions in systemic inflammatory markers observed with PIW treatment suggest that PIW has anti-inflammatory activity, consistent with evidence that targeting inflammation can mitigate atherosclerosis development [31]. Interestingly, the temporal pattern of cytokine reduction preceding macroscopic improvements in vascular morphology is consistent with the possibility that early anti-inflammatory modulation contributes to the vascular protective effects of PIW.

Extracellular matrix (ECM) degradation and remodeling are important determinants of plaque composition and stability. MMP-9 degrades collagen and other ECM components, which can contribute to plaque vulnerability, whereas its endogenous inhibitor TIMP-1 counterbalances proteolytic activity [32,33]. Elevated MMP-9 expression has been associated with plaque instability and adverse cardiovascular outcomes, whereas increased TIMP-1 expression is thought to restrain excessive ECM breakdown [34,35]. In the current study, PIW significantly reduced aortic MMP-9 and increased TIMP-1 levels, suggesting reduced proteolytic pressure within the vascular wall. These molecular changes may contribute to the observed attenuation of lesion-related histopathological features, including intimal thickening and lipid deposition. ET-1 is a potent vasoconstrictor and pro-inflammatory mediator implicated in endothelial dysfunction and atherosclerotic progression [36]. Elevated ET-1 levels promote oxidative stress, inflammation, and vascular smooth muscle proliferation [37]. The reduction in ET-1 levels observed with PIW is consistent with a broader vascular protective profile and may relate to improved endothelial function and reduced oxidative stress-related processes. The observed reductions in both serum and aortic ET-1 support the possibility of improved endothelial-dependent vasodilation, as ET-1-mediated ETA/ETB receptor signaling promotes vasoconstriction, oxidative stress, and vascular smooth muscle cell proliferation [37]. To confirm whether these molecular changes translate to measurable improvements in vascular reactivity, ex vivo aortic ring tension studies will serve as our next experiment.

Mechanistically, the coordinated changes observed in LOX-1/NF-κB–related protein signals, lipid-related indices, and inflammatory and proteolytic markers suggest that PIW may influence multiple interconnected processes involved in atherosclerosis. Such pleiotropic profiles are increasingly recognized as valuable in addressing complex chronic diseases that involve intertwined metabolic and inflammatory networks [31,38]. For comparison, atorvastatin was used as a reference intervention in this study. Atorvastatin is widely recommended as a first-line lipid-lowering therapy in contemporary clinical guidelines. As an HMG-CoA reductase inhibitor, it is used to lower atherogenic lipoprotein levels and reduce the risk of major cardiovascular events in specific patient populations [39]. Beyond LDL-C reduction, statins, including atorvastatin, have been reported to exert lipid-independent (“pleiotropic”) actions, including improved endothelial function and attenuation of inflammatory signaling, although the relative contribution of these effects in vivo may vary by context [40]. Consistent with these pleiotropic properties, atorvastatin in the present study was associated with improved serum lipid profiles, alleviated hepatic and vascular histopathological changes, and reduced inflammatory biomarkers and LOX-1/NF-κB-related protein signals in ApoE^−/−^ mice. However, statin therapy can be limited by tolerability and safety considerations, as atorvastatin labeling notes risks of myopathy and rare rhabdomyolysis, potential liver enzyme elevations and hepatic failure, increases in HbA1c and fasting serum glucose, and clinically relevant drug–drug or food interactions (e.g., CYP3A4 inhibitors and excessive grapefruit juice intake) that may increase atorvastatin exposure and adverse-event risk. In this context, PIW, as an edible bioactive ingredient, may represent a feasible functional food approach to support cardiometabolic health, potentially complementing established pharmacological strategies rather than serving as a therapeutic substitute; whether PIW provides additive or synergistic benefits when used alongside standard therapies remains to be evaluated. PIW exhibits a distinct long-chain alcohol composition compared with sugarcane policosanol, the current industry reference. Sugarcane policosanol typically contains ≥60% octacosanol, whereas PIW contains relatively higher proportions of hexacosanol and triacontanol, with a total hexacosanol and octacosanol content approximately three-fold greater than sugarcane and rice bran wax sources [19]. Compositional differences across policosanol sources can influence the bioactivity of policosanol [41], suggesting that insect-derived policosanol may offer distinct biological advantages. A direct comparative study at equivalent doses would definitively establish the relative potency of PIW compared to sugarcane policosanol, and this is planned as a priority in future research. Then, an intriguing observation is that the low-dose PIW group (75 mg/kg) demonstrated more sustained improvements in serum lipid profiles at week 8 compared with the high-dose group (150 mg/kg). This non-linear dose–response pattern may reflect hormetic mechanisms, whereby sub-saturating concentrations of octacosanol more efficiently activate AMPK and PPAR-α, promoting hepatic fatty acid β-oxidation and reducing de novo lipogenesis [18]. At higher doses, saturation of β-oxidation capacity or compensatory downregulation of cholesterol transporters (e.g., ABCA1, SR-B1) may partly attenuate net lipid-lowering efficacy. This dose-dependent variation warrants direct mechanistic investigation. Furthermore, emerging evidence links policosanol to modulation of the gut microbiota and gut–liver axis. Policosanol supplementation has been linked to greater abundance of *Akkermansia* and *Lactobacillus* species, which may strengthen gut barrier integrity and reduce systemic endotoxemia-driven vascular inflammation [42,43]. Whether PIW’s anti-atherosclerotic effects are partly mediated via this gut–liver pathway is a compelling hypothesis for future microbiome profiling studies.

The present study has several limitations. First, because PIW was administered concurrently with high-fat diet induction, our findings primarily reflect preventive effects rather than therapeutic regression of established plaques. Future studies should examine whether PIW can reverse advanced atherosclerotic lesions. Second, although LOX-1/NF-κB signaling was implicated, additional investigations into other relevant pathways, such as oxidative stress and lipid handling regulatory networks, are warranted. Third, the present study included only male ApoE^−/−^ mice. Biological sex is a critical variable in cardiovascular research, and estrogen has well-documented vascular protective, anti-inflammatory, and lipid-modulating properties [44]. The magnitude and underlying mechanisms of PIW’s anti-atherosclerotic effects may therefore differ between sexes, and future studies should include female mice across different hormonal states. Finally, while the ApoE^−/−^ mouse model recapitulates many aspects of human atherosclerosis, translation of these findings to human clinical contexts requires further validation. In conclusion, PIW attenuated diet-induced atherosclerosis in ApoE^−/−^ mice by improving lipid metabolism, suppressing inflammation, and modulating LOX-1/NF-κB-mediated signaling and ECM remodeling. These data support the potential of PIW as a functional food ingredient or nutraceutical for dietary management of atherosclerosis-related risk and provide a mechanistic framework to guide future translational efforts.

## 5. Conclusions

In summary, PIW demonstrated multi-faceted protective effects against diet-induced atherosclerosis in ApoE^−/−^ mice. Administration of PIW significantly reduced body weight, improved serum lipid profiles, and lowered systemic inflammatory markers, including IL-6, KC/CXCL1, TNF-α, and CRP. Histopathological analyses revealed attenuated hepatic and aortic tissue damage, reduced lipid deposition, and preservation of vessel wall architecture. At the molecular level, PIW modulated aortic ET-1, MMP-9/TIMP-1 balance, and LOX-1/NF-κB-related protein signals, suggesting coordinated effects on endothelial function, extracellular matrix remodeling, and inflammatory signaling. These findings support the notion that PIW exerts pleiotropic vascular protective actions, targeting interconnected metabolic and inflammatory pathways involved in atherosclerosis progression. In light of these findings, given its favorable safety profile as an edible bioactive, PIW may represent a promising functional food ingredient or nutraceutical to support supplement-based strategies for cardiovascular disease prevention and overall cardiovascular health. However, the present evidence derives from an ApoE^−/−^ mouse model employing a preventive design, and extrapolation to human therapeutic contexts requires caution.

## Data Availability

The original contributions presented in this study are included in the article. Further inquiries can be directed to the corresponding author.

## Abbreviations

The following abbreviations are used in this manuscript:

CRP	C-reactive protein
ET-1	Endothelin-1
H&E	Hematoxylin and eosin
HDL-C	High-density lipoprotein cholesterol
IL-6	Interleukin-6
IL-8	Interleukin-8
LDL-C	Low-density lipoprotein cholesterol
LOX-1	Lectin-like oxidized low-density lipoprotein receptor-1
MMP-9	Matrix metalloproteinase-9
NF-κB	Nuclear factor Κb
ox-LDL	Oxidized low-density lipoprotein
T-CHO	Total cholesterol
TG	Triglyceride
TIMP-1	Tissue inhibitor of metalloproteinase-1
TNF-α	Tumor necrosis factor-α
